# Genetic and peripheral biomarkers of comorbid posttraumatic stress disorder and traumatic brain injury: a systematic review

**DOI:** 10.3389/fneur.2025.1500667

**Published:** 2025-01-27

**Authors:** Kiriana Cowansage, Reshmi Nair, Jose M. Lara-Ruiz, Daniel E. Berman, Courtney C. Boyd, Tiffany L. Milligan, Daniel Kotzab, Dawn M. Bellanti, Lisa M. Shank, Maria A. Morgan, Derek J. Smolenski, Ida Babakhanyan, Nancy A. Skopp, Daniel P. Evatt, Marija S. Kelber

**Affiliations:** ^1^Psychological Health Center of Excellence, Defense Health Agency, Falls Church, VA, United States; ^2^Traumatic Brain Injury Center of Excellence, Defense Health Agency, Falls Church, VA, United States

**Keywords:** biomarker, PTSD, TBI, military, veteran, neurodegeneration, genetic, inflammation

## Abstract

**Background:**

Posttraumatic stress disorder (PTSD) commonly cooccurs with traumatic brain injury (TBI) in military populations and is a significant predictor of poor long-term outcomes; however, it is unclear to what extent specific biological variables are associated with comorbidity. This PROSPERO-registered systematic review evaluates the current body of literature on genetic and peripheral biomarkers associated with comorbid TBI and PTSD.

**Methods:**

Searches were conducted in four databases (PubMed, PsycInfo, PTSDPubs, Scopus). We included published studies examining differences in peripheral biomarkers among civilian, military, and veteran participants with both TBI and PTSD compared to those with TBI alone as well as, in some cases, PTSD alone and healthy controls. Data were extracted from included studies and evidence quality was assessed.

**Results:**

Our final analysis included 16 studies, the majority of which were based on data from active duty military and veteran participants. The results suggest that multiple gene variants are likely to contribute to the cumulative risk of PTSD comorbid with TBI. An elevated circulating level of the pro-inflammatory cytokine IL-6 was the most consistently replicated blood-based indicator of comorbid illness, compared to mTBI alone.

**Conclusion:**

Several genetic and protein markers of cellular injury and inflammation appear to be promising indicators of chronic pathology in comorbid TBI and PTSD. Additional research is needed to determine how such factors indicate, predict, and contribute to comorbidity and to what extent they represent viable targets for the development of novel diagnostic tools and therapeutic interventions.

## 1 Introduction

Posttraumatic stress disorder (PTSD) and traumatic brain injury (TBI) are burdensome and debilitating pathologies that occur at high rates in both civilian and military populations, though the latter community is disproportionately affected due to higher rates of exposure to psychologically traumatic events during combat (1). Data from over 3,000 veterans obtained through the National Health and Resilience in Veteran Study indicate a probable prevalence of PTSD around 16% (2), whereas the rate of TBIs in veterans deployed to Iraq and Afghanistan post-9/11 is estimated to be about 21% (3, 4). In the general population, the lifetime prevalences of PTSD and TBI are approximately 6 and 12%, respectively (5, 6).

The vast diversity of circumstances commonly surrounding injury type, mechanism, severity, and frequency represents a central impediment to understanding and predicting adverse long-term outcomes following TBI. Over 80% of all TBI cases are classified as mild (mTBI), diagnostically defined in Table 1 (7), and generally associated with high recovery rates (8). Nevertheless, a meaningful percentage of mTBI patients deteriorate over time, with PTSD representing one of its most common comorbidities (9). A time-to-event analysis of military participants with mTBI reported cumulative PTSD prevalence to be around 39% at 2 years post-injury and even higher in patients with moderate, severe, or penetrating TBIs (10). Moreover, data suggest that deployment-related injuries, a large percentage of which arise from blast exposure, are more likely to result in poor long-term neuropsychiatric outcomes (11, 12).

**Table 1:** Diagnostic criteria for mild TBI (mTBI).

Measure	Requirements for diagnosis
Glasgow coma score (GCS)	≥13
Loss of consciousness (LOC)	<30 min
Posttraumatic amnesia (PTA)	<24 h
Non-contrasted computed tomography (CT) scan	No skull fracture or visible intracranial injury*

^*^mTBI with evidence of structural intracranial injury on neuroimaging may be described as complicated mTBI.

In addition, because of the complexity and heterogeneity of both conditions, in conjunction with their high degree of symptom overlap, PTSD and mTBI are notoriously challenging to clinically differentiate. Common features of both pathologies include persistent patterns of hyperarousal, sleep disturbances, emotional dysregulation, cognitive impairments, diminished social and occupational functioning, and poor quality of life (6, 13). Individuals with both pathologies commonly experience a more severe symptom profile and worse long-term outcomes than those diagnosed with either condition alone (14). Among service members and veterans with combat-related injuries, the presence of PTSD after TBI is an even stronger predictor of long-term functioning than TBI severity (15).

Understanding the reasons for high rates of comorbidity is complicated by the wide range of circumstances surrounding the triggering event. Both conditions can be consequences of the same traumatic experience (16), and a history of TBI raises the risk of developing PTSD in both civilian and military samples (17, 18). A pre-TBI diagnosis of PTSD has also been found to predict poor post-TBI outcomes (13, 19).

Despite the high prevalence and medical impact of these disorders, there is limited efficient, economical, and accessible technology for screening and risk assessment, which may create barriers to early intervention. Moreover, many of the unique circumstances surrounding military service, such as deployment, special operations, combat exposure, and unrecognized blast injuries, can delay and complicate the diagnostic process (20, 21). Thus, developing and implementing standardized tools for identifying vulnerable individuals, accurately diagnosing comorbidity, and devising individualized treatment plans, would be of high clinical value to both military and civilian health care providers. Because many biological indicators of pathology, or biomarkers, can be readily detected in samples of blood or saliva, these accessible sources of biological data present promising avenues by which to gain insight into the underlying source of disease from the vantage point of genetic, epigenetic, transcriptional, and macromolecular systems. Once identified, such disease signatures can function as precision targets for drug, diagnostic, and screening tool development.

Both PTSD and TBI have already been the focus of sizeable bodies of work aimed at profiling candidate peripheral biomarkers in blood, cerebrospinal fluid (CSF), or by other methods of physiological sampling (22–25). Access to increasingly large genomic datasets has enabled genome-wide association studies (GWASs), which estimate the contributions of thousands of gene variants to the likelihood of a specific health-related outcome. GWASs have now produced summary statistics for both PTSD (26) and TBI (27). These values can be used to calculate polygenic risk scores (PRSs) for individual participants, providing a snapshot of cumulative genetic risk (28–30). There is also continued interest in studies of specific single nucleotide polymorphisms (SNPs), such as the apolipoprotein E (*APOE*) *ε4* gene variant, which may have outsized relevance to one or both conditions (31–33).

In addition, epigenetic mechanisms, such as DNA methylation and small non-coding RNAs, have received recent attention as potential mediators of experience-driven gene expression regulation that may shape the long-term trajectory of trauma-induced pathology (34–36). At the level of macromolecules, multiple protein-and lipid-based markers of inflammation, e.g., C-reactive protein (CRP), interleukin(IL)-6, tumor necrosis factor-alpha (TNFα); hypothalamic–pituitary–adrenal (HPA) axis dysregulation [e.g., corticotropin-releasing factor (CRF)]; neurodegeneration [e.g., ApoE, amyloid beta (Aβ), tau]; and cellular response to injury [e.g., glial fibrillary acidic protein (GFAP), vascular endothelial growth factor (VEGF), neurofilament light chain (NfL)] have been implicated in both acute and chronic consequences of traumatic experience (25, 37–39). These findings reveal striking points of convergence in multiple biochemical pathways, supporting the theory that one or more common mechanisms may explain the overlapping patterns of dysfunction observed in both PTSD and TBI.

Despite the advancements in biomarker research, there remains significant uncertainty about how this knowledge can be applied to the detection and differential diagnosis of comorbid TBI and PTSD. The goal of this systematic review is to evaluate the current state of the evidence for biomarkers that have a potential to inform this question. Specifically, we conducted a systematic review of studies of genetic and peripheral biomarkers implicated in comorbid TBI and PTSD. When possible, we highlight comparisons between comorbid and TBI-only participants, with the aim of identifying indicators that might help differentiate these complex pathologies.

## 2 Materials and methods

### 2.1 Overview

This systematic review (PROSPERO protocol number: CRD42023416360) was part of a larger project that originally sought to identify genetic, peripheral (i.e., measured outside the central nervous system), neuroanatomical, and neurophysiological biomarkers associated with TBI and PTSD comorbidity. For the purposes of this analysis, we did not include brain-based studies because a comprehensive systematic review was recently published on this topic (40).

### 2.2 Search strategy and eligibility criteria

A library specialist searched a combination of keywords related to biological markers, TBI, and PTSD in four databases (PubMed, PsycInfo, PTSDPubs, Scopus) from January 1994 to June 2024. Searches were limited to original research studies published in the English language, and they utilized controlled vocabulary mapping and explosion strategies when applicable. Variations of the complete PubMed search (Appendix 1) were conducted across all other databases. All eligible studies included at least one experimental group diagnosed with both conditions. Peer-reviewed experimental and observational studies were included if they compared measures of genetic or peripheral biomarkers in adults with comorbid TBI and PTSD to those in at least one TBI-only or PTSD-only group of adults (see Appendix 2 for full eligibility criteria).

### 2.3 Data screening and assessment of bias

The review team dually screened titles and abstracts, resolving disagreements through discussion and consensus. Full-text articles were obtained for records marked for inclusion at the title/abstract stage and were then dually screened using the same process. The team used a customized data extraction form to standardize the data collection process and ensure internal reviewer consistency. A single reviewer extracted study characteristics and results for each study, which were then reviewed and verified by a second reviewer.

Single raters used a customized version of the Quality in Prognostic Studies (QUIPS) (41) to judge the level of bias and methodological quality for each study across six domains including study participation, study attrition, prognostic factor measurement, outcome measurement, study confounding, and statistical analysis/reporting. We modified the QUIPS tool by providing more explicit definitions and examples of the prompting items for each domain, as well as adaptations of individual domains to improve their applicability to the topics of biomarkers, PTSD, and TBI. A second independent rater verified each assessment, and discrepancies were resolved through discussion or consultation with the research team as needed.

### 2.4 Effect size calculations

We were unable to conduct a meta-analysis because of the high level of heterogeneity across biomarker targets, methods of measurement, and study designs. When possible, we computed each study’s standardized mean difference (Cohen’s *d*) as the effect size estimate. We were particularly interested in understanding how the comorbid condition differed from either TBI or PTSD alone; however, nine studies did not have a PTSD-only group. Therefore, because the TBI-only group was the most consistently included across studies, we prioritized this comparison for the purposes of effect size calculations. When other conditions (e.g., PTSD-only and healthy controls) were included and relevant to interpreting the results of individual studies, we discuss those comparisons as well. Three cohort studies reported adjusted odds ratios between biomarker predictors and PTSD outcome (comorbid or TBI-only), and we converted the odds ratios to Cohen’s *d* (42). Six cross-sectional studies reported means, standard deviations (SD) or standard errors (SE) for biomarker measures comparing comorbid and TBI-only groups. Two studies provided results in graphical format only: median ± interquartile range (IQR) (43) and mean ± SE (44). We estimated these values by extracting data from high resolution images of the published bar graphs using the Plotdigitizer app (https://plotdigitizer.com/).

Two studies reported median and IQRs for biomarker measures comparing comorbid and TBI-only groups. For studies reporting median and IQR, we used the methods proposed by Wan et al. (45) and Luo et al. (46) to estimate the sample means and standard deviations for the biomarker outcome measures within each study. This proposed mean estimator is a weighted average of the mid-quartile range and median (see Appendix 3 for formula). Although these methods assume normal distributions, they provide a way to estimate effect sizes, enabling comparison across studies. For the 10 cross-sectional studies, we estimated standardized mean differences for biomarker outcomes comparing the comorbid and TBI-only groups. For the one randomized controlled trial (RCT) study, we computed standardized mean difference for the three biomarker outcomes used to compare the comorbid and TBI-only groups. Since the intervention tested in this study was not the focus of our study, we used only baseline data to examine differences between the two groups of interest.

## 3 Results

**Figure 1:**
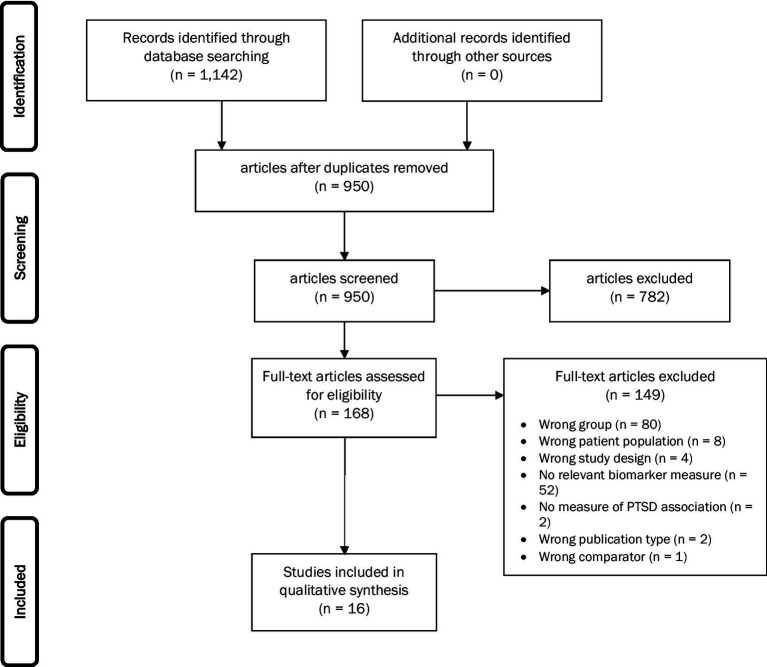
PRISMA flow diagram.

**Figure 2:**
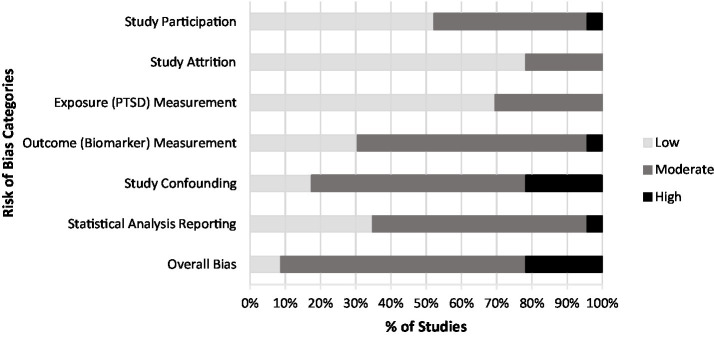
Risk of bias assessment of included studies.

Database searches yielded 1,142 references. After removing duplicates, the team screened 953 records, initially yielding 22 publications that met eligibility criteria. Of these, one group of three publications (43, 47, 48), one group of two publications (49, 50), and one group of four publications (18, 51–53) were identified as sharing common or potentially overlapping subject pools and were merged for the purposes of our analysis, resulting in a total of 16 studies. The PRISMA flow diagram (Figure 1) provides a detailed accounting of exclusions. The results of our risk of bias assessment identified only two publications rated as “low risk of bias” while most (*k* = 15) were rated as “moderate,” and *k* = 5 were rated as “high risk of bias” (Figure 2). The most common sources of bias were attributed to the use of self-report-based methods of diagnosing PTSD; the use of convenience sampling or other non-random participant recruitment methods; limited methodological information about the timing, conditions, or handling of biomarker collection; insufficient consideration of, and statistical adjustment for, covariates and confounding variables; and failure to report and/or explain the handling of missing data.

Among the 16 included studies, one (based on four publications) was a prospective cohort study, 14 were cross-sectional, and one was an RCT (Table 2). Fifteen studies explicitly recruited or were exclusively limited to service member and/or veteran participants. Most publications (*k* = 15) specifically focused on participants diagnosed with mTBI, as defined in Table 1 (7). Of the remaining seven publications, five included participants with all levels of TBI severity (52, 54–57), one included only participants with moderate–severe TBI (m-sTBI) (58), and one was limited to those with penetrating TBI (pTBI) (59). Studies were based on a total of 4,807 participants, of whom the majority were white and male (Table 2). Effect sizes and 95% confidence intervals (CI) are shown in Table 3 for all studies where means for both the TBI + PTSD and TBI-only groups were provided or could be calculated for the biomarker of interest.

**Table 2:** Study design and demographic characteristics.

First author, year	Study pop	Data source	Age, M(SD) or ME: IQR	% F	*% Wht*	Groups (N)					TBI severity	TBI DX Tool	PTSD DX criteria, tool/cutoff	Timing of study measures		
						*Total*	*TBI + PTSD*	*TBI only*	*PTSD only*	*HC*				*TBI*	*Bio*	*PTSD*
*Prospective Cohort*														*Time from TBI event*		
Kulbe, 2022	TC	TRACK-TBI	38: 26-55	32.9	77.2	1143	227	916	–	–	mTBI	GCS	DSM-5, PCL-5 ≥33	<24 h	<24 h	6 m
Nielson, 2017	TC	TRACK-TBI	43.3 (18.5)	28.5	71.5	586	338	248	–	–	All	GCS	DSM-IV, PCL-C	<24 h	<24 h	6 m
Stein, 2023	TC	TRACK-TBI	44.6 (18.2)	35.3	100.0	714	116	598	–	–	mTBI	GCS	DSM-5, PCL-5 ≥33	<24 h	<24 h	6 m
Winkler, 2017	TC	TRACK-TBI	40.5 (16.3)	40.0	69.9	93	28	65	–	–	mTBI	GCS	DSM-IV, PCL-C	<24 h	<24 h	6 m
*Cross-sectional*														*Mean time from TBI to study enrollment*		
Emmerich, 2016; Huguenard, 2020; Nkiliza, 2023^1^	SM	-	26.8 (7.0)	0.0	74.2	120	13	21	34	52	mTBI	BTBIS	DSM-IV, PCL-M ≥35	<3 y	–	–
Gill, 2014	SM	-	33.7 (8.1)	2.7	67.3	110	20	13	3	42	All	WARCAT	DSM-III-R, PCL-M ≥50	>6 m	–	–
Guedes, 2021	SM/V	LIMBIC-CENC	37: 31-49	11.0	ND	144	45	71	-	28	mTBI	OSU TBI-ID/ VCU rCDI	DSM-5, PCL-5 ≥38	Lifetime	–	–
Hanas, 2019	V	-	43.3 (8.4)	3.0	ND	65	33	12	10	10	All	Self-Report	BSSQ≥6	5-14 y	–	–
Ho, 2014	V	WRIISC	34.0 (10.0)	12.1	72.4	58	6	7	11	34	mTBI	VAT-BIST/ RBANS	ND	ND	–	–
Kinzel, 2020; Umminger, 2023^2^	All	INTRuST	35.4 (12.3)	36.1	74.1	147	41	32	6	68	mTBI	INTRuST mTBISI	DSM-IV/5, MINI, PCL-5, SCID	Lifetime	–	–
Marquardt, 2018	V	NG & VAHCS	32.3 (8.1)	7.3	91.1	123	34	22	28	39	mTBI	MN-BEST	DSM-IV-TR, CAPS, SCID	4-5 y	–	–
Nielsen, 2019	V	–	ND	ND	ND	87	26	21	3	37	mTBI	CIQ	DSM-IV, PCL-C ≥50	ND	–	–
Pardini, 2012	V	VHIS	58. (2.6)	0.0	100	113	31	82	-	-	pTBI	ND	DSM-IV-TR, CAPS, SCID	36–39 y	–	–
Pattinson, 2019	SM	15y LS	34.2 (10.1)	7.5	71.6	102	21	63	-	18	mTBI	OSU TBI-ID	DSM-IV-TR, PCL-C	4–5 y	–	–
Ramos-Cejudo, 2021	V	–	32.5 (7.2)	7.9	57.8	230	43	70	53	64	All	OSU TBI-ID	DSM-IV/5, CAPS	13y	–	–
Smith, 2022	SM/V	–	37: 31-45 (TBI+PTSD); 36: 29.5-43.5 (TBI)	18.7	76.4	123	62	61	–	–	All	OSU TBI-ID	DSM-IV, PCL-C ≥50	4–8 y	–	–
Weiner, 2023	V	VHA NDS	69.5 (4.5)	0.7	84.4	289	94	43	81	71	m-sTBI	OSU TBI-ID	DSM-IV, SCID, CAPS ≥40 (PTSD); CAPS ≥30 (PTSD+TBI)	Lifetime	–	–
Xu, 2018	V	CVC	32.8 (6.9)	0.0	41.9	489	52	158	44	235	mTBI	OSU TBI-ID	DSM-5, CAPS	ND	–	–
*RCT*														*Time to baseline measurement*		
Weaver 2018	SM/V	–	32.8 (7.3)	1.0	ND	71	31	30	–	–	mTBI	Structured Interview	DSM-IV, SCID	<5y	–	–

^1Studies led by Emmerich, Huguenard, and Nkiliza are based on measures derived from the same subject pool and will be considered together for the purposes of this review. 2Studies led by Umminger and Kinzel are based on measures derived from the same subject pool and will be considered together for the purposes of this review. 15y LS, 15-Year Longitudinal TBI Study; Bio, Biomarker; BSSQ, Breslau Short Screening Questionnaire; BTBIS, Brief TBI Screen; Ctr, Center; CSF, Cerebrospinal fluid; Civ, Civilian; CAPS, Clinician Administered PTSD Scale; CIQ, Clinical Injury Questionnaire; CVC, Cohen Veteran Center; CRF, Corticotropin releasing factor; Dx, Diagnostic; DSM-IV, Diagnostic and Statistical Manual of Mental Disorders, 4th Edition; DSM-5, Diagnostic and Statistical Manual of Mental Disorders, 5th Edition; EV, Extracellular vesicle; F, Female; GCS, Glascow Coma Score; HC, Healthy Control; h, Hours; HPA, Hypothalamic pituitary adrenal; INTRuST, Injury and Traumatic Stress Clinical Consortium; IQR, Interquartile range; INTRuST mTBISI, INTRuST mTBI Screening Instrument; LIMBIC-CENC, Long Term Impact of Military Brain Injury Consortium—Chronic Effects of Neurotrauma Consortium; M, Mean; ME, Median; mTBI, Mild traumatic brain injury; MINI, Mini-International Neuropsychiatric Interview; m-sTBI, Moderate–severe TBI; m, Months; NG, National Guard; ND, Not described; OSU TBI-ID, Ohio State University TBI Identification Method; PCL-C, PTSD Checklist-Civilian Version; PCL-M, PTSD Checklist-Military Version; pTBI, Penetrating TBI; Pop, Population; RCT, Randomized controlled trials; RBANS, Repeatable Battery for Neuropsychological Testing; SM, Service Members; SCID, Structured Clinical Interview for DSM-IV-TR; SD, Standard deviation; TC, Trauma center; TRACK-TBI, Transforming Research and Clinical Knowledge in TBI; V, Veterans; VAHCS, Veterans Affairs Health Care System; VHA NDS, Veterans Health Administration National Data Systems; VAT-BIST, Veteran Traumatic Brain Injury Screening Tool; VHIS, Vietnam Head Injury Study; WRIISC, War Related Illness and Injury Study Center; WARCAT, Warrior Administered Retrospective Casualty Assessment Tool; W, Weeks; Wht, White; y, Years.^

**Table 3:** Biomarker targets, methods of analysis, and summary of results.

First author, year	Level of biochemical analysis	Sample type	Biomarker target(s)	Function	Overall correlation of marker with PTSD+TBI (PTSD presence or severity)	Effect sizes (*d* [95% CI]) calculated for TBI + PTSD vs. TBI-only
Prospective cohort						
Kulbe, 2022	Protein	Plasma; Serum	GFAP hsCRP	Astrocytic injury response Inflammation	log(GFAP): ↓ log(hsCRP): nca	log(GFAP): −0.09 [−0.15, −0.03] log(hsCRP): 0.06 [−0.01, 0.12]
Nielson, 2017	Genetic (SNP)	Whole Blood, Plasma	*ANKK1* (rs1800497, rs4938016, rs11604671); *COMT Val^158^Met* (rs4680); *DRD2* (rs6277) *5HT2AR* (rs6311) *BDNF* (rs6265) *APOE ε2* (rs7412) and *ε4* (rs429358) *OPRM1* (rs1799971) *BCL2* (rs17759659) *PARP1* (rs3219119)	Dopamine modulation Serotonin signaling Neuronal plasticity Neurodegeneration Mu opioid signaling Regulation of apoptosis DNA Repair	*ANKK1*: ↑; *COMT*: ↑; *DRD2*: ↑ (all trends) *5HT2AR*: nca *BDNF*: nca *APOE*: nca *OPRM1*: nca *BCL2*: nca *PARP1* (A/T): ↑	See text
Stein, 2023	Genetic (Genome-Wide)	Whole Blood	PRS for PTSD	Polygenic risk of PTSD	PRS: ↑	0.32 [0.19, 0.44]
Winkler, 2017	Genetic (SNP)	Whole Blood	*COMT Val^158^Met* (rs4680)	Dopamine signaling	*COMT Val^158^Met*: ↓	−0.63 [−1.22, −0.02]
Cross-sectional						
Emmerich, 2016; Huguenard, 2020; Nkiliza, 2023	Genetic (SNP) Protein Lipid	Plasma	*APOE ε4* (rs429358) Aβ40; Aβ42; Aβ38 GFAP FABP3 Lipid Panel	Neurodegeneration (SNP) Neurodegeneration (protein) Astrocytic injury response (protein) Lipid regulation (protein) Lipid regulation/metabolism (lipid)	*APOE* [within ε4-]^1^: tot TG/DG: ↑; tot Cer ↑ Aβ38: nca Aβ40: nca Aβ42: nca GFAP: nca FABP3: nca PL (multiple classes): ↓ tot PC: ↓; MUFA-containing PC and PI species: ↓; tot TG and DG: ↑; AA-derived oxylipins: ↑; oxidized PUFAs: ↑; AA- and DHA-containing ethanolamides (multiple species): ↑	Aβ38: −0.12 [−1.01, 0.77] Aβ40: −0.44 [−1.16, 0.28] Aβ42: −0.19 [−0.89, 0.51] FABP3: −0.17 [−0.93, 0.60] GFAP: −0.13 [−0.85, 0.59]
Gill, 2014	Protein	Plasma	IL-6; CRP	Inflammation	IL-6: ↑ CRP: nca	IL-6: 0.88 [0.15, 1.61] CRP: 0.65 [−0.06, 1.37]
Guedes, 2021	Protein Epigenetic	Plasma; EV	IL-10; IL-6; CRP; TNFα Aβ40; Aβ42; NfL; t-tau VEGF miRNA Panel	Inflammation (protein) Neurodegeneration (protein) Cellular injury/repair (protein) Neurodegeneration (epigenetic: miRNA)	IL-6 (EV): ↑ IL-6 (plasma): nca^2^ IL-10 (EV and plasma): nca TNFα (EV): nca TNFα (plasma): ↓ Aβ40 (EV): ↑ Aβ40 (plasma): nca Aβ42 (EV and plasma): nca t-tau (EV and plasma): nca VEGF (EV and plasma): nca NfL (EV and plasma): ↑ hsa-miR-374a-3p: nca^2^ hsa-miR-139-5p: ↑^3^ hsa-miR-1185-1-3p: ↑ hsa-miR-425-5p: ↓ hsa-miR-3190-3p: ↑ hsa-miR-1277-3p: nca^2^ hsa-miR-204-5p: nca hsa-miR-372-3p: nca hsa-miR-509-3-5p: nca hsa-miR-615-5p: nca hsa-miR-375: nca hsa-miR-3196: nca	IL-6 (EV): 0.55 [0.17, 0.93] IL-6 (plasma): 0.57 [0.19, 0.95]^2^ IL-10 (EV): −0.19 [−0.56, 0.19] IL-10 (plasma): −0.15 [−0.53, 0.22] TNFα (EV): −0.17 [−0.55, 0.20] TNFα (plasma): −0.40 [−0.77, −0.02] Aβ40 (EV): 0.48 [0.10, 0.86] Aβ40: (plasma): 0.29 [−0.08, 0.67] Aβ42 (EV): 0.18 [−0.20, 0.55] Aβ42: (plasma): 0.31 [−0.06, 0.69] t-tau (EV): −0.07 [−0.44, 0.31] t-tau (plasma): 0.23 [−0.15, 0.60] VEGF (EV): 0.12 [−0.25, 0.49] VEGF (plasma): −0.24 [−0.61, 0.14] NfL (EV): 0.86 [0.47, 1.25] NfL (plasma): 0.67 [0.29, 1.06] hsa-miR-[]: 374a-3p: −0.63 [−1.02, −0.25]^2^ 139-5p: 0.25 [−0.13, 0.62]^3^ 1185–1-3p: 0.59 [0.20, 0.97] 425-5p: −0.40 [−0.78, −0.03] 3190-3p: 0.44 [0.06, 0.82] 1277-3p: −0.43 [−0.81, −0.05]^2^ 204-5p: −0.10 [−0.47, 0.27] 372-3p: −0.09 [−0.46, 0.29] 509–3-5p: −0.10 [−0.47, 0.28] 615-5p: 0.09 [−0.29, 0.46] 375: −0.13 [−0.50, 0.25] 3196: 0.00 [−0.37, 0.37]
Hanas, 2019	Protein	Serum	Protein Panel	Multiple	See text	See text
Ho, 2014	Epigenetic	Plasma	snoRNA Panel	Neurodegeneration	ACA48: ↓; U35: ↓; U55: ↓; U83A: ↓	See text
Kinzel, 2020; Umminger, 2023	Lipid	Serum	Allo; Preg	Neurosteroid	Allo: ↓; Preg: nca	Allo: −0.33 [−0.80, 0.13] Preg: 0.17 [−0.30, 0.63]
Marquardt, 2018	Cardiovascular Electrodermal		HRD SCR	Cardiovascular Physiological arousal	HRD:↑ SCR: ↑	HRD: 0.29 [−0.31, 0.88] SCR: 0.76 [0.09, 1.43]
Nielsen, 2019	Protein Epigenetic	Plasma	ApoE protein *APOE* promoter methylation	Neurodegeneration (protein) Neurodegeneration (epigenetic: DNA methylation)	ApoE: ↑ See text	ApoE (ε4+)^1^: −0.25 [−1.37, 0.88] ApoE (ε4-)^1^: 0.43 [−0.25, 1.11]
Pardini, 2012	Genetic (SNP)		*FAAH* SNP Panel	Lipid regulation	*FAAH* SNP rs2295633 [vmPFC lesion(−) only]: ↑	See text
Pattinson, 2019	Protein	Plasma	t-tau; Aβ42;	Neurodegeneration	t-tau: ↑ Aβ42: nca	t-tau: 0.62 [0.11, 1.12] Aβ42: 0.05 [−0.44, 0.55]
Ramos-Cejudo, 2021	Protein	Serum	CRF	HPA axis	CRF: ↓	−0.95 [−1.35, −0.55]
Smith, 2022	Protein	Plasma	IL-10; IL-8; IL-6; IL1RA; CRP; TNFα VEGF p-tau	Inflammation Cellular injury/repair Neurodegeneration	IL-6: ↑ IL-8: ↑ IL-10: ↑ TNFα: ↑ IL1RA: nca CRP: nca; VEGF: nca p-tau: nca	IL-6: 0.64 [0.27, 1.00] IL-8: 0.77 [0.40, 1.14] IL-10: 0.47 [0.11, 0.83] TNFα: 1.16 [0.78, 1.54] IL1RA: 0.11 [−0.24, 0.47] CRP: 0.29 [−0.07, 0.64] VEGF: −0.06 [−0.42, 0.29] p-tau: −0.12 [−0.47, 0.24]
Weiner, 2023	Protein	CSF	p-tau; t-tau; Aβ42	Neurodegeneration	p-tau: nca t-tau: nca Aβ42: nca	p-tau: −0.16 [−0.52, 0.21] t-tau: −0.17 [−0.53, 0.19] Aβ42: 0.10 [−0.26, 0.46]
Xu, 2018	Other	Plasma	Mixed Panel		See text	See text
RCT						
Weaver, 2018	Cardiovascular		HRV	Cardiovascular	Baseline HRV (HF): nca Baseline HRV (LF): ↑^3^ Baseline HRV (VLF): ↑^3^	HF: −0.27 [−0.78, 0.23] LF: 0.44 [−0.07, 0.95]^3^ VLF: 0.50 [−0.01, 1.01]^3^

^1^Results stratified by APOE ε4 carrier status, as indicated by ε4+ or ε4-. ^2^Overall association with PTSD symptoms was not conclusive, but effect size calculation suggests a group difference between the TBI + PTSD and TBI-Only groups. ^3^Overall association suggests a positive correlation with PTSD symptoms, but effect size calculation suggests no conclusive difference between the TBI + PTSD and TBI-Only groups. AA, Arachidonic acid; Allo, Allopregnanolone; Aβ, Amyloid beta; ApoE, Apolipoprotein E; DHA, docosahexaenoic acid; DG, Diglycerides; FABP3, Fatty acid binding protein 3; GFAP, Glial fibrillary acidic protein; HRD, Heart rate deceleration; HRV, Heart rate variability; HF, High frequency; hsCRP, High sensitivity C-reactive protein; LF, Low frequency; MUFA, Monounsaturated fatty acid; NfL, neurofilament light chain; nca, No conclusive association; PC, Phosphatidylcholine; PL, Phospholipid; p-tau, phosphorylated tau; PARP1, Poly (ADP-ribose) polymerase 1; PRS, Polygenic risk score; PUFA, Polyunsaturated fatty acid; Preg, Pregnanolone; SCR, Skin conductance response; SNP, Single nucleotide polymorphism; tot, Total; t-tau, Total tau; TG, Triglycerides; VEGF, Vascular endothelial growth factor; VLF, Very low frequency.

### 3.1 Prospective studies of post-TBI PTSD

All four cohort publications (52, 53, 60, 61) were based on prospective longitudinal data from participants in the multicenter Transforming Research and Clinical Knowledge in TBI (TRACK-TBI) study, following their admission to a Level 1 Trauma Center for treatment of a head injury. Publications were inclusive of all participant backgrounds (civilian, military, and veteran) and recruited patients from multiple United States hospitals. Nielson et al. (52) and Winkler et al. (53) obtained data from San Francisco General Hospital (CA), University of Pittsburgh Medical Center (PA), and University Medical Center Brackenridge (Austin, TX). Stein et al. (60) and Kulbe et al. (51, 61) obtained data from across 18 centers between February 2014 and August 2018. Based on the group and demographic information provided, we were unable to determine to what extent subject overlap may have occurred. One publication also made use of the National Institutes of Health-National Institute of Neurological Disorders and Stroke TBI common data elements (TBI-CDE) initiative, which aimed to assemble and standardize a comprehensive dataset from TBI patients enrolled in the TRACK-TBI Pilot study (52). Participants received TBI diagnoses, provided blood samples within 24 h of injury, and were evaluated for the presence of PTSD 6 months later. As described in the following sections, the general objective of these studies was to investigate the extent to which one or more gene or protein of interest predicted PTSD following TBI.

#### 3.1.1 Genetic factors

Three distinct computational methods were used to investigate either monogenic or polygenic risk factors for PTSD in the 6 months after a TBI. Winkler et al. (53) tested the importance of a single gene variant; Nielson et al. (52) applied a machine learning technique to concurrently investigate the role of 12 SNPs with hypothesized functional implications for TBI and neuropsychiatric health; and Stein et al. (60) derived PRSs using summary statistics from prior GWASs of PTSD (26) to estimate the cumulative risk conferred by thousands of gene variants.

At the level of individual SNPs, two publications evaluated the effect of the *COMT Val^158^Met* gene variant on six-month outcomes (52, 53). Winkler et al. (53) found that, after adjusting for pre-existing psychiatric disorders and illicit drug use, individuals with mTBI who carried the *Met^158^* variant were less likely than their *Val/Val* counterparts to develop PTSD 6 months later (adjusted odds ratio: 0.32, 95% CI: 0.11, 0.97; *d* = −0.63, 95% CI: −1.22, −0.02). In contrast, the findings by Nielson et al. (2017) suggest the opposite pattern. After applying a combination of general linear models and machine learning-driven topological data analysis (TDA) to explore relationships among variables contained within the TBI-CDE, the authors reported enrichment of *COMT Val^158^Met*, along with five other gene variants (*DRD2*, *PARP1*, and three *ANKK1* SNPs), in a subset of mTBI participants with PTSD diagnoses and worsening Glasgow Outcome Scale—Extended (GOS-E) scores from three to 6 months post-injury. Further TDA mapping of TBI severity, PTSD Checklist-Civilian Version (PCL-C), and GOS-E outcome measures revealed that among patients lacking visible damage on CT scans, a time-dependent increase in functional impairment was predicted by the presence of the A/T SNP of *PARP1*, a gene associated with cellular stress and injury response signaling pathways.

#### 3.1.2 Acute post-injury protein levels

One publication investigated whether acute blood protein levels of GFAP and high sensitivity C-reactive protein (hsCRP), measured within 24 h post-injury, can function as long-term predictors of PTSD (51, 61). The authors reported that although GFAP levels were higher in TBI participants compared to uninjured controls, GFAP levels among TBI patients were negatively correlated with the severity of PTSD symptoms 6 months later, as measured by the PCL-5 (Table 3). In contrast, acute post-injury hsCRP levels were not associated with PTSD among TBI patients.

### 3.2 Cross-sectional analyses of biofluid-based markers

Across the 13 cross-sectional studies of blood-or CSF-derived biomarkers associated with comorbid PTSD and TBI, we observed substantial variability in the interval of time from injury to clinical assessment, both within and between studies (Table 2). As described in the following sections, these studies focused on a range of genetic, epigenetic, and macromolecular indicators of neurodegeneration (*k* = 6); inflammation (*k* = 3); lipid trafficking and metabolism (*k* = 3); and HPA axis dysfunction (*k* = 1).

#### 3.2.1 Alzheimer’s disease-related neurodegeneration

Six cross-sectional studies examined blood-or CSF-based markers of neurodegeneration that have established involvement in the pathophysiology of Alzheimer’s Disease (AD) and/or TBI. Of the studies that analyzed known biomarkers of AD-related pathology (e.g., ApoE, tau, Aβ), three either stratified or adjusted their analyses for the presence of the *APOE ε4* SNP (43, 58, 62), while three did not control for genotype (57, 63, 64). All participants in these studies were either military service members or veterans, though participants differed widely in the circumstances of, and time elapsed since their most recent TBI.

Four studies compared protein levels of one or more Aβ isoforms measured in plasma, extracellular vesicles, or CSF, with Aβ42 being the most frequently measured variant; however, none of these studies reported conclusive differences between TBI + PTSD and comparison groups (Table 3) (43, 58, 63, 64). Similar negative findings were reported from measures of Aβ38 and Aβ40 (43, 63), although Huguenard et al. (2020) saw increased Aβ42/Aβ40 ratios among mTBI-only and TBI + PTSD compared to the PTSD-only participants, and Guedes et al. (63) found a weak correlation between levels of Aβ40 in extracellular vesicles and PCL-5 scores among combat-exposed veterans and service members with histories of mTBI.

Levels of total tau (t-tau) and phosphorylated tau (p-tau) were measured in three studies of service members and veterans with remote histories of TBI; however, results were inconsistent. Pattinson et al. (64) found elevated plasma t-tau concentrations with a moderate effect size but large confidence interval (*d* = 0.62, 95% CI = 0.11, 1.12) among service members with a current PTSD diagnosis plus a history of TBI in the last 4–5 years. In contrast, Weiner et al. (58) found no differences in CSF levels of either total t-tau or p-tau among PTSD-diagnosed veterans with a lifetime history of TBI. Likewise, Smith et al. (57) saw no differences in plasma p-tau levels between groups. Only one study examined levels of ApoE, the direct protein product of the *APOE* gene (62). Here, the authors tested samples of plasma obtained from veterans with TBI + PTSD, either condition alone, or neither condition, finding that concentrations of ApoE were positively associated with PTSD symptom severity.

#### 3.2.2 Epigenetic mechanisms of gene regulation

To understand the mechanism of the observed correlation of ApoE with PTSD symptoms, Nielson et al. (62) went further to investigate whether DNA methylation of specific sites on the *APOE* gene might account for altered ApoE protein expression in participants with more severe PTSD, either with or without the *APOE ε4* SNP. They reported that circulating levels of plasma ApoE levels were positively associated with *APOE* methylation at CpG −775 and negatively associated with methylation at CpG-877; however, there were no conclusive differences in circulating plasma ApoE between TBI + PTSD and TBI-only participants among *ε4* carriers (*d* = −0.25, 95% CI: −1.37, 0.88) or non-carriers (*d* = 0.43, 95% CI: −0.25, 1.11).

Two additional studies of epigenetic regulatory mechanisms examined changes in small non-coding RNAs hypothesized to modulate gene expression pathways linked to neurodegeneration. Guedes et al. (63) identified four micro RNAs (miRNAs) isolated from extracellular vesicles—hsa-miR-139-5p, hsa-miR-1185-1-3p, hsa-miR-3190-3p, and hsa-miR-425-5p—that were correlated with PCL-5 scores. Linear regression further supported a specific association between PCL-5 scores and hsa-miR-139–5p. In addition, hsa-miR-1185-1-3p and hsa-miR-3190-3p were upregulated in TBI + PTSD versus TBI-only participants with moderate effect sizes coupled with large confidence intervals (1185–1-3p: *d* = 0.59, 95% CI: 0.20, 0.97; 3,190-3p: *d* = 0.44 95% CI: 0.06, 0.82), whereas hsa-miR-374a–3p, hsa-miR-1277-3p, and hsa-miR-425-5p were conversely downregulated (374a-3p: *d* = −0.63, 95% CI: −1.02, −0.25; 1,277-3p: *d* = −0.43, 95% CI: −0.81, −0.05; 425-5p: *d* = −0.40, 95% CI: −0.78, −0.03). Meanwhile, Ho et al. (65) identified four small nucleolar RNAs (snoRNAs) that were downregulated in blood samples from veterans with mTBI + PTSD compared to participants with PTSD only. Using the Unweighted Pair Group Method with Arithmetic Mean agglomerative unsupervised hierarchical clustering, the authors then classified veterans with PTSD in the presence or absence of TBI, reporting the ability of these snoRNAs to differentiate comorbid TBI + PTSD from PTSD alone (sensitivity = 1.00, 95% CI:[0.51–1.00]; accuracy = 0.82; and specificity = 0.72, 95% CI: [0.39–0.93]). The sensitivity and specificity of the classification procedure were assessed using receiver operating characteristic (ROC) analysis.

#### 3.2.3 Cellular injury and repair

Three studies investigated known markers of cellular injury and repair, including GFAP, NfL, and VEGF (43, 57, 63). All participants in these studies were service members and/or veterans, either with or without distant/lifetime histories of TBI and in the presence or absence of PTSD. Huguenard et al. (43) did not observe differences in GFAP between TBI + PTSD and any comparison conditions. Guedes et al. (63) found that in both plasma and extracellular vesicles from service members with TBI + PTSD, NfL was elevated compared to TBI-only samples (extracellular vesicles: *d* = 0.86, 95% CI: 0.47, 1.25; plasma: *d* = 0.67, 95% CI: 0.29, 1.06). Moreover, both extracellular vesicle and plasma levels of NfL were associated with PCL-5 scores in a linear regression model that adjusted for total number of mTBIs and times since last TBI. Neither Smith et al. (57) nor Guedes et al. (63) described consistent differences in blood levels of VEGF among comorbid service members and veterans compared to any of the other groups.

#### 3.2.4 Lipid regulation and metabolism beyond APOE

Three studies (reported in five publications) investigated connections between TBI + PTSD and readouts of lipid regulation and metabolism with undetermined or indirect links to *APOE* (43, 47, 48), individual gene variants (59), and correlates of neurosteroid levels (49, 50). As previously noted, three of the included publications were based on repeated analyses of the same 120-person study population (43, 47, 48), while an additional two publications were based on multiple analyses of the same 147-person study population (49, 50).

Using liquid chromatography-mass spectrometry, Emmerich et al. (48) found that blood plasma levels of multiple classes of phospholipid species were reduced by 24–40% in service members with TBI + PTSD compared to healthy controls, and more severe PTSD (PCL-Military Version scores ≥44) was associated with lower levels of multiple phospholipid classes. A similar pattern of total phospholipid reduction was also observed in PTSD-only and TBI-only groups compared to healthy controls. Both Huguenard et al. (43) and Nkiliza et al. (47) used a similar approach to analyze blood lipid and lipid metabolite levels as a function of *APOE ε4* carrier status in samples from the same group of study participants. The former measured levels of triglycerides, diglycerides, and several other lipid subtypes, revealing widespread alterations in multiple triglycerides and diglycerides species, including elevated levels of total triglycerides and diglycerides, specifically in TBI + PTSD subjects *without* the ε4 allele compared to all other groups. The latter focused on levels of two classes of lipid metabolites, oxylipins and ethanolamides, reporting changes in multiple lipid species that varied as both functions of comorbidity and genotype.

Pardini et al. (2012) investigated lipid dysregulation from the perspective of a set of SNPs identified in the fatty-acid amide hydrolase (*FAAH*) gene among Vietnam war veterans with combat-related penetrating TBIs (pTBIs). Specifically, the authors asked whether any of seven known FAAH SNPs were associated with an increased likelihood of comorbid PTSD diagnosis, and whether any such genetic effect(s) interacted with the presence or absence of pTBI-related lesions to the ventromedial prefrontal cortex. Only one of the seven SNPs (rs2295633) was associated with an increased prevalence of PTSD, and this higher rate of TBI + PTSD comorbidity was only observed in the subset of individuals *without* pTBI-related damage to the ventromedial prefrontal cortex. No links between any FAAH SNPs and PTSD were observed in pTBI patients with ventromedial prefrontal cortex lesions.

Kinzel et al. (49) and Umminger et al. (50) used data collected from a common set of study participants (comprising both civilians and service members) to investigate whether serum levels of two circulating neurosteroids, allopregnanolone and pregnenolone, differ among participants with TBI + PTSD versus those with either or neither condition. In addition, they asked whether any observed differences in neurosteroids were moderated by either brain-wide cortical thickness (49) or fractional anisotropy (50), as measured by magnetic resonance and diffusion tensor imaging. Kinzel et al. (49) observed that, compared to healthy controls, mTBI + PTSD was associated with a modest but inconclusive reduction in serum allopregnanolone (*d =* −0.33, 95% CI: −0.80, 0.13); however, this difference was not replicated in Umminger et al. (50). No differences in allopregnanolone were observed among other experimental groups, and no groups differed in levels of pregnenolone. Compared to healthy control and mTBI-only participants, comorbidity was also associated with multiple areas of reduced cortical thickness, which correlated positively with both serum allopregnanolone and pregnenolone (49). In addition, Umminger et al. (50) observed a positive association between serum levels of allopregnanolone and whole brain fractional anisotropy, reflective of white matter microstructure integrity, and this effect was enhanced in individuals with TBI + PTSD.

#### 3.2.5 Inflammation

Three studies investigated differences in blood levels of key inflammatory markers in service members and veterans with TBI + PTSD (54, 57, 63). Markers tested included IL-6, IL-8, IL-10, TNFα, and CRP. All three analyses reported at least marginally elevated levels of IL-6 in individuals with TBI + PTSD compared to one or more control conditions. Guedes et al. (63) observed elevated levels of extracellular vesicle IL-6 in TBI + PTSD compared to mTBI-only service members and veterans, yielding a moderate effect size (*d* = 0.55, 95% CI: 0.17, 0.93). This difference also manifested as a weak correlation with PTSD severity, determined using scores on the PCL-5. Gill et al. (54) also investigated the role of inflammatory markers in post-deployment military personnel with histories of TBI and/or PTSD. After controlling for age, body mass index, and medications, concentrations of IL-6 were elevated in participants with high comorbidity (TBI + PTSD + depression) compared to those with no more than one service-related disorder, and TBI alone was not associated with increased IL-6 (*d* = 0.88, 95% CI: 0.15, 1.61). Finally, Smith et al. (57) tested plasma levels of IL-6 in service members and veterans with a lifetime history of TBI, either with or without a current diagnosis of PTSD. After adjusting for body mass index, number of TBIs, Combat Exposure Scale score, and time since last injury, the authors found that, compared to TBI participants without symptoms of PTSD, those with PTSD symptoms had higher levels of IL-6, as well as elevated IL-8, IL-10 and TNF-*α*, with moderate to large effect sizes reported for all four markers (IL-6: *d* = 0.64, 95% CI: 0.27, 1.00; IL-8: *d* = 0.77, 95% CI: 0.40, 1.14; IL-10: *d* = 0.47, 95% CI: 0.11, 0.83; TNFα: *d* = 1.16, 95% CI: 0.78, 1.54). The latter three results diverge from Guedes et al. (63), however, who did not observe any group differences in levels of IL-10 or TNF-α (Table 3) and who did not measure IL-8. No study observed differences in CRP across any group comparison.

#### 3.2.6 HPA Axis dysregulation

Only one study examined biomarkers directly associated with HPA axis dysregulation in TBI + PTSD. In this case, Ramos-Cejudo et al. (56) examined serum CRF levels in veterans with a remote history of TBI, finding reduced levels of circulating CRF in comorbid and PTSD-only participants compared to healthy and TBI-only comparators (*d* = −0.95, 95% CI: −1.35, −0.55). Furthermore, CRF levels were negatively correlated with PTSD symptom severity, as measured by scores on the Clinician-Administered PTSD Scale for DSM-5.

#### 3.2.7 Other multi-target panels

Two studies examined complex multi-target panels that could not be readily described in terms of individual candidate biomarkers or specific mechanistic pathways. Both studies were geared toward the development of diagnostic tools that might help distinguish overlapping phenotypic profiles associated with PTSD and TBI, both diagnosed alone and in combination. To this end, Xu et al. (66) investigated whether metrics conventionally tested on routine bloodwork panels could be used to inform the development of a low-cost diagnostic tool. The authors used random forest classification models to attempt to predict TBI and PTSD groupings based on a stepwise inclusion of features. Although no single variable was independently sufficient to differentiate specific diagnostic categories, the authors reported that together, measures of insulin, homeostatic model assessment for insulin resistance, aspartate aminotransferase, neutrophil counts, and triglycerides were the most important features for discriminating TBI + PTSD from healthy controls (area under the curve = 0.74, accuracy = 0.74, sensitivity = 0.64, specificity = 0.77).

Likewise, Hanas et al. (55) asked whether a large panel of interconnected peptide/protein targets could inform the development of a serum analytical platform using mass spectrometry in conjunction with the “leave one out serum sample cross-validation” (LOOCV) methodology. If successful, the purpose of this tool would be to differentiate distinct disease characteristics associated with post-TBI complications and comorbidities. Participants included service members and veterans with either deployment related TBIs in the last 5–14 years (with or without PTSD and depression comorbidity) or no diagnosed traumatic injury/illness. The authors reported the identification of discriminatory mass peaks indicative of distinct molecular profiles associated with TBI + PTSD versus TBI-only and healthy control participants; however, additional research is needed to fully interpret these results and assess the clinical utility of this method of analysis.

### 3.3 Physiological measures of cardiovascular health and arousal

Two studies investigated cardiovascular and psychophysical health metrics in the context of comorbid PTSD and TBI. The first, a cross-sectional study in post-deployment veterans, investigated psychophysical responses to emotionally arousing pictures to determine if post-blast mTBI (with or without PTSD) led to altered sensitivity to aversive combat-related content. Measures included startle electromyography, skin conductance response, and heart rate deceleration (44). These data revealed an increase in SCR (*d* = 0.76, 95% CI: 0.09, 1.43) and inconclusive changes in heart rate deceleration among participants with TBI + PTSD when compared directly to those with TBI-only (*d* = 0.29, 95% CI: −0.31, 0.88); however, parallel changes in skin conductance response and heart rate deceleration were observed in both the PTSD-only and TBI + PTSD participants, irrespective of TBI history, suggesting that the PTSD diagnosis (across TBI conditions) was the principle driver of these effects.

The second study was an RCT, aimed at investigating physiological response to hyperbaric oxygen treatment in military participants with either TBI alone or comorbid with PTSD (67). Because evaluating interventions was outside the scope of this review, and this was the only RCT that met our criteria for inclusion, we limited our analysis exclusively to differences in baseline heart-rate variability measures between the two clinically distinct populations (TBI-only and TBI + PTSD). We focused on this time point, rather than post-intervention outcomes, because it provided the most direct and unaltered measure of intrinsic biological differences between the two groups of interest. The results of this comparison revealed that both very low frequency and low frequency heart-rate variability were elevated in TBI + PTSD individuals compared to TBI-only; however, the confidence interval included the possibility of null findings (very low frequency: *d* = 0.50; 95% CI = -0.01, 1.01; low frequency: *d* = 0.44, 95% CI = -0.07, 0.95). In contrast, high frequency heart-rate variability changed modestly in the opposite direction but did not conclusively differ between groups (*d* = −0.27, 95% CI = -0.78, 0.23).

## 4 Discussion

PTSD is among the most common and debilitating complications of trauma-related TBI; yet there is still a relatively small literature exploring the biological dynamics underpinning their association and comorbidity. This systematic review provides a comprehensive analysis of 16 studies aimed at evaluating peripherally sampled biological indicators of pathology associated with comorbid TBI and PTSD. Although there remain significant hurdles to implementing these findings in clinical practice, we synthesize the current literature on this topic and identify several promising avenues of investigation that warrant future research. In particular, the pro-inflammatory cytokine, IL-6, is among the only targets identified across multiple studies as a persistent indicator of comorbid pathology that may remain elevated in the bloodstream even years after initial injury. This protein is rapidly synthesized as part of both acute and chronic immune system responses to many types of infection and injury (68), making it a viable target for innovations in risk assessment and screening tools, yet also potentially limiting its utility as a disease-specific marker. In the brain, chronic and dysregulated expression of IL-6 is implicated in the disease progression of a range of neurological and psychiatric disorders. In the central nervous system, persistent IL-6 activity during illness or injury is thought to contribute to neurodegeneration by increasing inflammation; disrupting homeostatic and cellular repair mechanisms; triggering chronic engagement of glial cells; and altering properties of the blood–brain barrier (69). Several antibody-based drugs targeting IL-6 or its receptors are already in clinical trials or have received approval for the treatment of autoimmune disorders (70). While these developments may suggest a causal role for IL-6 in many pathological processes, the ubiquitous nature of this molecular pathway and its benign involvement in normal immune response mechanisms also potentially make IL-6 a challenging target for mechanistic studies of specific neurobiological conditions. Given the small number of studies conducted on this topic and the high likelihood of polytrauma-related influences from multiple confounding factors in this unique patient population, the implications of the current findings in the context of TBI and PTSD must be interpreted with caution.

Biomarker research has emerged in the last decade as a promising new frontier in the evolution of precision medicine, yet there continues to be a need for more rigorous longitudinal studies of its applications and predictive utility. Access to affordable and accurate blood-based biomarker testing, particularly among those served by the Military Health System, which has historically suffered from shortages in specialized mental health care services, can potentially improve risk prediction, reduce stigma surrounding mental health diagnoses, facilitate the differential diagnosis of complex conditions, inform treatment strategies, and support individualized care. Such tests have already been clinically implemented in a range of medical specialties. For example, in cardiovascular medicine and endocrinology, comprehensive bloodwork panels for metabolic disease markers are increasingly used to identify sources of preventable risk before the onset of chronic conditions, like atherosclerosis and diabetes (71). In neurology, screening for known indicators of AD can allow individuals to make proactive lifestyle changes and begin treatment years before noticeable signs of cognitive impairment (72, 73); and life-saving next generation genomic sequencing by liquid biopsy is increasingly used in the field of oncology to develop highly targeted treatment plans and detect recurrence, sometimes weeks to months before metastatic disease is observed by traditional imaging techniques (74).

Despite these medical advances, standardized clinical applications of biomarkers to the prevention, diagnosis, and treatment of brain injury and neuropsychiatric disease remain elusive, in large part because of the immense underlying complexity associated with the mechanisms of human behavioral pathologies. While innovations in blood-based diagnostic tools, such as those measuring acute trauma-induced levels of S100 calcium-binding protein B (S100B), carboxyl-terminal hydrolase-L1 (UCH-L1), and GFAP can potentially help expedite screening at the time a TBI occurs (75), such tests must be conducted at short timepoints post-injury. Despite evidence supporting their potential applications as predictors of long-term outcomes, biomarkers such as GFAP have not yet been standardized for widespread clinical implementation as prognostic tools to guide long-term care (76–78).

Genetic contributions to disease have, until recent years, been studied through the lens of single genes, using loss-and gain-of-function-based studies to investigate their impact on specific biochemical pathways. This approach has had limited efficacy for tracing the sources of complex phenotypes associated with polygenic neuropsychiatric disorders. Recent advances in computational methods for analyzing data from GWASs, however, have enabled the development of procedures for calculating aggregated disease risk based on the contributions of many genes (28). Promising new research supports the idea that these PRSs could eventually be implemented to estimate individual susceptibility for both TBI and PTSD (79). Although the literature on this subject is very recent, published evidence to date provides initial support for the use of PRSs in predicting risk of comorbid PTSD after TBI (60).

Despite the limitations of single-gene analyses, some individual variants may warrant more focused investigation (80). For example, the *APOE* gene, which encodes the lipid trafficking protein, ApoE, has critical multi-system functions in both brain and cardiometabolic health. Carriers of the *APOE ε4* SNP incur an approximately three-fold increase per allele in lifetime odds of developing late-onset AD, making it the single largest genetic source of risk (81). The functional consequences of this gene variant are attributed to the expression of a less effective form of the ApoE protein, resulting in the increased pathological aggregation of amyloid beta (Aβ)—a hallmark feature of AD (72, 81). In the context of TBI, the ApoE protein appears to be important for mechanisms of neuronal repair and recovery following TBI (82). Several studies have found that veterans carrying *APOE ε4* in conjunction with a probable history of TBI and/or PTSD have more severe symptoms, worse long-term neuropsychiatric outcomes, and higher rates of cognitive decline than their non-ε4 counterparts (83–85). Abnormalities in lipid trafficking, metabolism, and regulation have likewise been implicated in the pathophysiology of PTSD (86), further bolstering the theory that *APOE* and other lipid regulatory mechanisms may be influential in both conditions.

Likewise, the *COMT* gene, which encodes the catechol-O-methyltransferase (COMT) enzyme, may also have specific implications for comorbid TBI and PTSD. COMT catalyzes the degradation of catecholamines and is a critical regulator of dopamine turnover in the prefrontal cortex (87). Substitution of valine (Val) for methionine (Met) at this locus leads to a reduction in COMT enzymatic activity, resulting in enhanced dopaminergic neurotransmission (88, 89). Some previous studies have found that carriers of *COMT Val158Met* have improved cognitive function in some domains but also heightened levels of arousal and anxiety (90)—traits hypothesized to be associated with worse post-injury outcomes and elevated risk of PTSD (91). Nevertheless, data on this subject remain conflicting and inconclusive (92), and some findings discussed in this systematic review in fact suggest a possible protective effect of *Met158* on risk of post-TBI PTSD, underscoring the need for additional research based on larger sample sizes with rigorously controlled experimental designs.

Beyond the genome itself, epigenetic mechanisms of gene regulation, via DNA methylation, histone modification, and the actions of small non-coding RNAs, are increasingly recognized as critical transducers of environmental stimuli into biologically interpretable signals that can mediate crosstalk between heritable factors and traumatic experience (24, 35, 36). Here, we document several intriguing but inconclusive findings that support further investigation of the contributions of miRNAs, snoRNAs, and DNA methylation in the control of genes involved in neurodegeneration, inflammation, cellular response to injury, and HPA axis function that may be relevant to the development of comorbid TBI and PTSD.

In addition, both neurodegeneration and inflammation signaling pathways, which are tightly interrelated, have already been implicated separately in the mechanisms of pathology underpinning PTSD and TBI (93, 94). Several of these indicators, detectable in both blood and CSF, have increasingly established associations with both neurological and neuropsychiatric diseases, including TBI and PTSD (22). Among individuals with a history of TBI, recent evidence suggests that biomarkers implicated in inflammatory processes may persist at elevated levels for extended durations, even weeks to months post-injury, making them potentially promising predictors of poor long-term prognosis and risk for adverse outcomes, like post-concussive syndrome and PTSD (94–96). Consistent with this idea, this review found the most consistently replicated effect to be a pattern of elevated levels of the pro-inflammatory cytokine, IL-6, among individuals with PTSD diagnosed at distant timepoints following TBI. Fully understanding how data from such blood-based protein measures can be effectively harnessed in clinical settings to track the evolution of chronic pathologies will necessitate innovations in technology for economical high-throughput screening; and larger studies using patients with more consistently tracked diagnostic histories and injury chronologies.

### 4.1 Limitations and future directions

This systematic review only included studies published in English, thus studies published in other languages may have been omitted. The interpretability of findings discussed in these predominantly cross-sectional studies was undercut by their high degree of heterogeneity, both across studies and within subjects, preventing us from meta-analyzing the results. Participants varied substantially in their time from TBI to clinical evaluation; the intervals separating injury from PTSD onset; and the timing of biomarker measurement with respect to both TBI and PTSD—factors that were further complicated by the wide range of circumstances, characteristics, and confounding risk factors surrounding the TBIs prior to development of PTSD. The small body of literature on this topic offers only early insights into the complex interplay between these conditions, and future studies are needed that more extensively document and control for many other critical variables, such as the number, mechanism, and severity of past TBIs; presence of other psychiatric comorbidities; history of adverse childhood experiences; and record of underlying physical conditions that might augment baseline levels of inflammatory and neurodegenerative markers independent of TBI and PTSD.

The generalizability of our conclusions was also constrained by the demographic characteristics of the study samples, since females and non-White participants were significantly underrepresented, and most of the existing data come from males (range: 60–100%) who identified as White, Caucasian, or of European descent (range: 42–100%). This lack of gender, ethnic, and racial diversity in biological datasets limits our ability to extend insights from these findings to broader populations. Epidemiological data show that females are diagnosed with PTSD at higher rates than males, and multiple biological factors, including differences in genetics, hormones, and brain connectivity are likely to contribute. For example, a recent analysis of genetic linkage between PTSD and testosterone revealed a negative correlation between testosterone levels and PTSD in men, suggesting that genetic variations underpinning differences in sex hormones may also contribute to risk of PTSD (97). The need to capture more representative biological samples in studies of human disease should be an important consideration in the design of future studies—especially given that 17.5% of active duty service members are female and 31.2% identify as non-White (98).

## 5 Conclusion

At present, there is limited published evidence and no consensus on the use of specific biomarkers to predict PTSD following TBI. Nevertheless, studies using a range of methodologies to investigate multiple genetic, epigenetic, and macromolecular indicators offer insights into disease mechanisms that may eventually translate into clinically useful tools to augment the efficacy of risk assessment and diagnostic methods. To date, the most consistently replicated result is that increased levels of the pro-inflammatory cytokine, IL-6, remained associated with comorbidity even years after initial injury. This finding stands in contrast with several studies of other commonly tested markers (e.g., CRP and Aβ42) that consistently failed to produce group effects associated with comorbidity. Other promising avenues of investigation include the use of microRNA profiling to better understand gene–environment interactions and PRSs based on GWASs to evaluate individual disease susceptibility. All results discussed in this review warrant additional study and replication to determine the most robust targets for innovation in this rapidly advancing field of research.

## Data Availability

The original contributions presented in the study are included in the article/supplementary material, further inquiries can be directed to the corresponding author.

## Author disclaimer

The views expressed in this manuscript are those of the authors and do not necessarily represent the official policy or position of the Defense Health Agency, Department of Defense, or any other U.S. government agency. This work was prepared under Contract #W81XWH-21-F-0322 with DHA Contracting Office W81XWH and, therefore, is defined as U.S. Government work under Title 17 U.S.C.§101. Per Title 17 U.S.C.§105, copyright protection is not available for any work of the U.S. Government. For more information, please contact dha.ncr.j-9.mbx.phcoe-contact-us@health.mil.

## Appendix

### Appendix 1. PubMed Search String

(“Stress disorders, post-traumatic”[MeSH] OR “post-traumatic stress disorder”[tiab] OR “post traumatic stress disorder”[tiab] OR “posttraumatic stress disorder”[tiab] OR PTSD[tiab]) AND (“Brain injuries, traumatic”[MeSH] OR “traumatic brain injur*”[tiab] OR “TBI”[tiab] OR “mTBI”[tiab]) AND (Biomarkers[MeSH] OR “genetic markers”[MeSH] OR biomarker*[tiab] OR biological[tiab] OR physiological[tiab] OR immun*[tiab] OR genetic[tiab] OR blood[tiab] OR serum[tiab] OR molecular[tiab] OR inflamm*[tiab] OR histology[MeSH] OR histology[tiab] OR neuroimaging[tiab] OR neurophysiological[tiab] OR neuroanatomical[tiab])

## Appendix 2. Eligibility Criteria

Studies were included if they met all the following criteria:

1. Peer-reviewed article.
2. Experimental or observational study design (i.e., prospective or retrospective cohort; case–control; cross-sectional).
3. Published in the English language.
4. Published between 1994 and 2024.
5. Adult (≥ 18 years of age) population with a comorbid TBI (of any severity) and PTSD condition diagnosed by a validated diagnostic PTSD scale.
6. Comparison group(s) of healthy, PTSD-only, and/or TBI-only participants.
7. Measurement of at least one genetic or peripheral biological marker of a pathogenic process/response.

## Appendix 3. Formula for Mean Estimation

Estimate of sample mean = $w\;\left(\frac{q_{1}+q_{3}}{2}\right)+\left(1-w\right)m$ and

Estimate of standard deviation = $\frac{\left(q_{3}-q_{1}\right)}{2\Phi^{-1}\;\left(\frac{\left(0.75n-0.125\right)}{n+0.25}\right)\text{.}}$

where $q_{1}$ = first quartile of the data, m =median of the data, $q_{3}$ = third quartile of data, $w=0.7+\frac{0.39}{n}$ is the weight assigned to the mid-quartile range $\left(\frac{q_{1}+q_{3}}{2}\right)$ , and remaining weight 1−w is assigned to the median m , n = sample size, and $\Phi^{-1}\left(z\right)$ is the inverse function of $\Phi \left(z\right)$ , or equivalently, the upper *z*th percentile of the standard normal distribution computed by the command “qnorm(z)” in statistical software R.
